# Effect of Traditional Chinese Medicine Bufei Granule on Stable Chronic Obstructive Pulmonary Disease: A Systematic Review and Meta-Analysis Based on Existing Evidence

**DOI:** 10.1155/2020/3439457

**Published:** 2020-02-07

**Authors:** Yihua Fan, Xinyan Wen, Qiang Zhang, Fangyuan Wang, Qing Li, Xinju Li, Yi Guo

**Affiliations:** ^1^Tianjin University of Traditional Chinese Medicine, Tianjin 301617, China; ^2^The First Teaching Hospital of Tianjin University of TCM, Tianjin 300193, China; ^3^Department of Oncology, Army Medical Center of PLA, ChongQing 400042, China

## Abstract

This systematic review and meta-analysis aimed at evaluating the effect of traditional Chinese medicine (TCM) Bufei granule on stable chronic obstructive pulmonary disease (COPD). We retrieved data from PubMed, Web of Science, EMBASE, the Cochrane Central Register of Controlled Trials, CNKI, Wanfang, and WeiPu (VIP) for studies focusing on whether the TCM Bufei granule would be effective in treating stable COPD. No language restriction and blinding were used. All trials involved were examined based on the standards of the Cochrane Handbook, and Review Manager 5.3 software was applied for analyzing data. We included four studies involving 599 patients with stable COPD. When compared to placebo treatment, TCM Bufei granule intervention exhibited improvement in the forced expiratory volume in one second (FEV_1_) (standardized mean difference (SMD) = 0.70; range, 0.50–0.91; *I*^2^ = 0%), forced vital capacity (FVC) (SMD = 0.43; range, 0.23–0.62; *I*^2^ = 0%), FEV_1_ percentage of predicted value (FEV1%) (SMD = 0.57; range, 0.38–0.76; *I*^2^ = 4%), and FEV_1_/FVC (SMD = 0.69; range, 0.50–0.87; *I*^2^ = 0%). There was a statistically significant difference in St George's Respiratory Questionnaire scores between the TCM Bufei granule and placebo treatments (SMD = −1.29; range, −2.32 to −0.26, *I*^2^ = 97%). None of the studies reported any adverse events. Therefore, TCM Bufei granule intervention could help in improving the lung function and quality of life in patients with stable COPD.

## 1. Introduction

Chronic obstructive pulmonary disease (COPD) is a common health issue worldwide [1–3] due to its increasing prevalence, financial burden, and associated morbidity. According to the projection for 2020, it will be among the top three diseases in the world responsible for deaths [4]. In China, COPD will be responsible for the deaths of 65 million people in 30 years from 2003, mainly due to tobacco smoking and use of solid fuel [5]. Although evidence-based treatment has been recommended for dealing with COPD, including short-acting bronchodilators, long-acting bronchodilators (LABAs), inhaled glucocorticosteroids, and low-dose and slow-release theophylline [6], these treatments are inevitably associated with several side effects [7, 8]. These unwanted side effects could cause some people with COPD to explore other treatment options. Therefore, it is essential to design better strategies and interventions for COPD.

Traditional Chinese medicine (TCM) has been the treatment for COPD for many years. In China, many COPD patients are treated with TCM modalities daily. Studies have shown the positive effects of using TCM drugs in stable COPD [9–14]. However, they lacked reasonable research scheme design and were limited to one medical centre or were without placebo control.

In the TCM theory, COPD affects the lung in the initial phase of the pathophysiology as well as the progression of the disease. COPD ultimately causes Ben-root deficiency Biao-branch excess syndrome, which is considered as Qi deficiency of the lungs, spleen, and kidneys, along with phlegm retention and blood stasis [15, 16]. The pathogenesis of COPD can be explained by the deficiency of antipathogenic Qi and excess of pathogenic Qi. Thus, the idea of the treatment is to focus on strengthening vital Qi, dispelling blood stasis, and resolving phlegm. Bufei granule has been developed according to the treatment principle that aims at enhancing the immune functioning of the lungs and ZangFu organs. This protection can prevent disease progression in COPD patients [15]. Several clinical trials [15, 17–19] have indicated the therapeutic effect of TMC Bufei granule in COPD patients with respect to improving the patient's wellbeing and lung function. However, there is no design that systematically assesses the quality of these trials, and their results remain controversial and inconclusive. The objective of this review was to compare the positive impact of TCM Bufei granule intervention and placebo intervention in adults with stable COPD.

## 2. Materials and Methods

### 2.1. Search Strategy

Randomized controlled studies reported through September 9, 2019, were systematically searched in PubMed, EMBASE, the Cochrane Central Register of Controlled Trials, Web of Science, CNKI, Wanfang, and VIP databases. The search terms mainly included COPD, chronic obstructive pulmonary disease, Bufei granule, and Bufei keli ([Supplementary-material supplementary-material-1]). Other reference materials and relevant articles were also searched. All literature review was carried out by the two investigators (Yihua Fan and Xinyan Wen). When disagreements arose, a third investigator (Qiang Zhang) was engaged until an agreement was reached.

### 2.2. Inclusion and Exclusion Criteria

Inclusion criteria were as follows:

1. Randomized controlled studies on Bufei granule treatment of stable COPD
2. Stable COPD patients diagnosed as per the criteria for diagnosis, management, and prevention of COPD as reported by the Global Initiative for Chronic Obstructive Lung Disease (GOLD) [20]; according to the pulmonary function test (GOLD guide application of bronchiectasis drugs), FEV_1_ ≥30% and <80% predicted value and FEV_1_/FVC < 70% diagnosed as stable moderate to severe COPD (grade 2 GOLD, GOLD level 3) and stable COPD patients with stable symptoms such as cough, sputum production, or breathlessness
3. The intervention of the treatment group included conventional therapy and Bufei granule
4. Conventional therapy and placebo granule were used to treat the control group
5. Outcomes of our study included St George's Respiratory Questionnaire (SGRQ) score [21], FEV_1_, FVC, FEV_1_/predicted value (FEV_1_%), and FEV_1_/FVC

Exclusion criteria were as follows:

  Expert comment, conference report, republished literature, or case reports were excluded

### 2.3. Study Selection

Two investigators (Yihua Fan and Xinyan Wen) independently selected the studies using Endnote X7 software based on the predetermined criteria. First, they eliminated duplication among the different databases from the initial aggregated search results. Second, by reviewing the titles of studies and their abstracts, seemingly irrelevant studies were excluded. Third, two investigators screened the full texts of the potential related studies and excluded unqualified studies. Both investigators independently cross-checked the selection results of the studies. Any disagreements during the study period were discussed, and a third investigator (Qiang Zhang) was approached for consulting and decision making if the problem was not resolved.

### 2.4. Data Extraction

Following the literature selection, the two investigators (Yihua Fan and Xinyan Wen) separately used an extraction sheet to extract data from the studies, including the first author's name, year of publication, sample size, participants' average age, intervention measures, time of intervention, and outcome indicators. We also interacted with the authors by sending e-mails or calling on the phone, in order to gather further information that was not included in the literature. For continuous outcomes, standard deviations and means were extracted.

### 2.5. Quality Evaluation

Quality of research studies was assessed independently by two investigators (Yihua Fan and Xinyan Wen) using the risk of bias evaluation for randomized clinical trials in the systematic Cochrane Reviews. Evaluation items included random sequence generation, allocation concealment, blinding of participants and personnel, blinding of outcomes assessment, incomplete outcome data, selective reporting, and other biases. The third investigator (Qiang Zhang) resolved the discrepancies. The meta-analysis was performed in accordance with the Preferred Reporting Items for Systematic Reviews and Meta-Analyses (PRISMA) guidelines ([Supplementary-material supplementary-material-1]) [22].

### 2.6. Statistical Analyses

All statistical analyses were carried out using Review Manager 5.3 software (The Nordic Cochrane Centre, The Cochrane Collaboration, 2014). The results were presented as the standardized mean difference (SMD) with the 95% confidence interval (95% CI). The clinical heterogeneity of the included studies was measured by the *χ*^2^ test. If *I*^2^ was <50%, the heterogeneity among studies was assumed to be small, and we used the fixed-effects model for data analysis. If heterogeneity was detected (*I*^2^ ≥50%), we chose the random-effects model or used only qualitative descriptions. The stability and reliability of the meta-analysis were evaluated by sensitivity analysis. Egger's and Begg's tests were used for the evaluation of potential publication bias.

## 3. Results

### 3.1. Search Results

**Figure 1:**
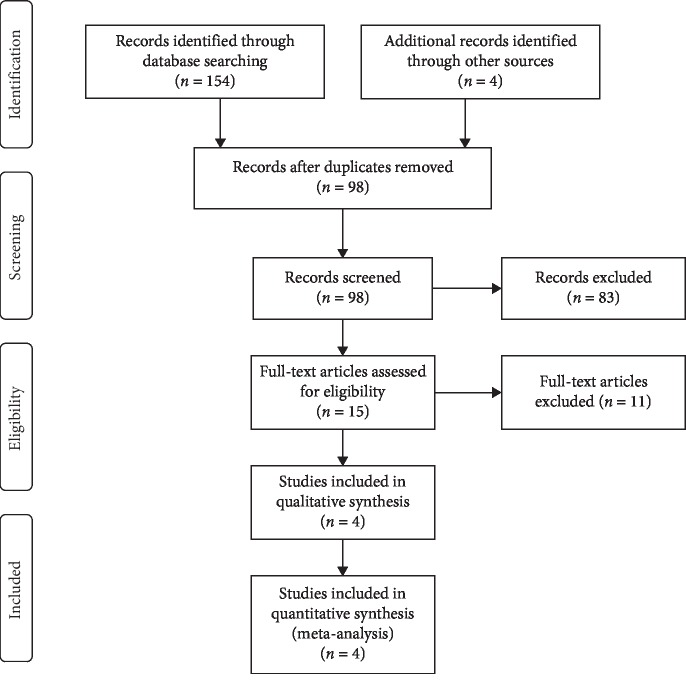
The flow diagram.

Our search scheme yielded 154 citations, and additional four citations were identified by examining reference lists of relevant kinds of literature. After removing duplicates and reviewing abstracts, 15 full texts were obtained and four of them [15, 17–19] were part of the final analysis (Figure 1).

### 3.2. Characteristics and Methodological Quality of the Eligible Studies

**Figure 2:**
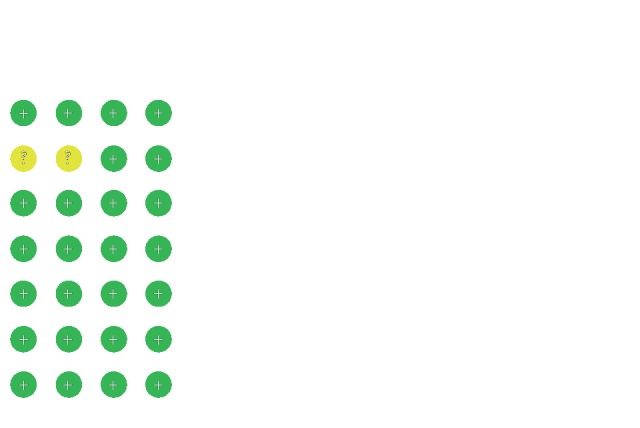
Risk of bias summary.

The characteristics of the four studies are described in Table 1, and they included 599 patients [15, 17–19]. All studies were conducted in China. Three studies lasted for 12 weeks [15, 17, 19], while one lasted for 30 days [18]. The quality and risk of bias assessment of the involved studies are described in Figure 2.

### 3.3. Synthesis of Results

#### 3.3.1. Pulmonary Function

**Figure 3:**
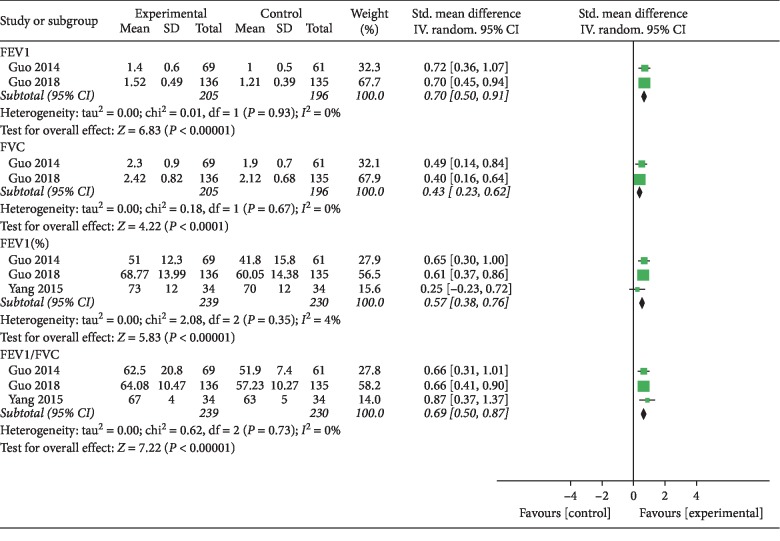
Meta-analysis of pulmonary function.

Two studies [15, 17] reported FEV_1_ data. Total meta-analysis showed that the effect of Bufei granule was better than that of the placebo (SMD = 0.70; range, 0.50–0.91; *I*^2^ = 0%). Two studies [15, 17] used FVC as the outcome. The pooled analysis revealed a statistically significant improvement in the FVC with TCM Bufei granule as compared to that of the placebo treatment (SMD = 0.43; range, 0.23–0.62; *I*^2^ = 0%). Three studies [15, 17, 19] reported FEV_1_% data. Total meta-analysis showed that Bufei granule had a better effect than the placebo treatment (SMD = 0.57; range, 0.38–0.76; *I*^2^ = 4%). Three studies [15, 17, 19] used FEV_1_/FVC as the outcome, and the pooled analysis showed a statistically significant improvement in the FEV_1_/FVC with TCM Bufei granule than that of the placebo treatment (SMD = 0.69; range, 0.50–0.87; *I*^2^ = 0%). Here, Figure 3 shows the efficacy of Bufei granule in stable COPD patients.

### 3.4. St George's Respiratory Questionnaire Score

**Figure 4:**
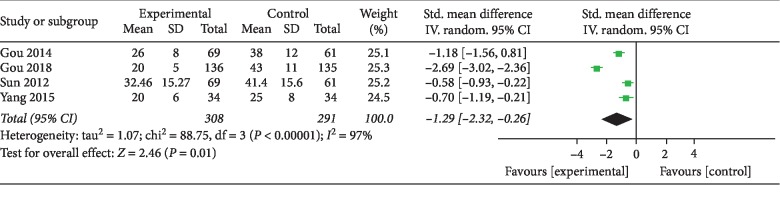
Meta-analysis of SGRQ score.

Four studies [15, 17–19] used the SGRQ score, and a reduction in the SGRQ score implied an improvement in the patient's wellbeing. The TCM Bufei granule treatment caused a statistically significant improvement in health-related quality of life as compared to the placebo (SMD = −1.29; range, −2.32 to −0.26, *I*^2^ = 97%). The result of the SGRQ score analysis is presented in Figure 4.

### 3.5. Adverse Events

None of the studies reported any adverse events.

### 3.6. Sensitivity Analysis

The stability and reliability of the meta-analysis were evaluated by sensitivity analysis. When the study by Guo et al. [15] was excluded for the SGRQ score, the result was reversed, thus indicating that the study result was not stable. Other outcome measures were stability and reliability.

### 3.7. Publication Bias

Begg's funnel plot ([Supplementary-material supplementary-material-1]) and Egger's test were performed to evaluate the publication bias of the included studies. The results showed no statistically significant difference (Begg's test *P*=0.497; Egger's test *P*=0.338).

## 4. Discussion

Previously, smoking cessation was considered the only way to treat the COPD progression because it caused a reduction in FEV_1_ and mortality rates. However, the pharmacotherapy for COPD has significantly improved in the past decade [23, 24] due to the accessibility of inhalational corticosteroids [25], fixed combinations of inhaled corticosteroids, long-acting *β*2-agonists (LABAs) [7], and long-acting anticholinergic or muscarinic antagonists [26], which have improved the patients' treatment outcomes. TCM has been used to treat COPD for many years, especially in China. TCM believes that the main pathogenesis of COPD in the stable stage is a Qi deficiency in the lungs, spleen, and kidneys. This is because of the long period of attack, long exogenous pathogenic factors in the lung, lung Qi damage, phlegm turbidity and heat stasis, and damage to vital-qi. Therefore, the main treatment in the stable stage is aimed at strengthening the vital-Qi.

In the stable phase of COPD, the pathogenesis of COPD is the deficiency of antipathogenic Qi and excess of pathogenic Qi. Its treatment should be based on strengthening vital-qi, eliminating blood stasis, resolving phlegm to relieve asthma, and improving the function of viscera to enhance the defensive capacity, thereby effectively preventing further development of the disease. Bufei granule is a decoction of the Bufei decoction in “Qianjin Yaofang for Emergency Preparedness” and Jinshui Liujun decoction in “Jingyue Quanshu New Eight Arrangements,” composed of Dangshen, Shudihuang, Shanzhuyu, Mahuang, Chenpi, etc. [17]. Its main functions include invigorating the spleen, nourishing the kidneys, invigorating the lungs, removing blood stasis, resolving phlegm, and relieving asthma. Modern studies [15, 17, 27] have shown that Bufei granule can downregulate the levels of proinflammatory cytokines tumor necrosis factor-*α* (TNF-*α*), interleukin-8 (IL-8), and transformation growth factor-1*β* (IL-1*β*) in the alveolar lavage fluid; upregulate the expression of anti-inflammatory cytokines interleukin-4 (IL-4) and interleukin-10 (IL-10); inhibit the inflammatory response of lung tissue; and exert a clinically therapeutic effect. Li et al. [10] showed that Bufei granule could decrease the level of serum soluble ST2 by increasing the levels of serum interleukin-33 (IL-33), IL-4, and IL-10, which in turn enhances the anti-inflammatory ability of the body. Therefore, by analyzing the mechanism of Bufei granule in modern experiments, it can be inferred that it has important research value in the treatment of COPD. Our study collected the latest randomized, double-blind, and placebo-controlled trials and conducted a comprehensive quantitative analysis.

This systematic review demonstrates the present evidence regarding Bufei granule in the treatment of COPD. Bufei granule along with conventional medical treatment could improve lung function and the quality of life in COPD patients better as compared to placebo plus conventional medicine. The FEV_1_, FVC, FEV_1_%, and FEV_1,_/FVC are important indicators for evaluating lung function. The decrease in FEV_1_ indicates poor lung function, while increase in FEV_1_ indicates improvement in COPD pulmonary function. However, single evaluation of FEV_1_ cannot evaluate the postdiastolic response in patients accurately. Therefore, FEV_1_, FVC, FEV_1_%, and FEV_1,_/FVC parameters were used to evaluate the pulmonary function better. The efficacy of Bufei granule is according to the recommended treatment standard of GOLD, which can alleviate current symptoms and risks [22]. The SGRQ score not only measures the patient's condition change, but also assesses the patient's psychological state. It also has a good correlation with FEV_1_, FEV_1_%, FEV_1_/FVC, and clinical symptoms of patients and further reflects their social activities, daily life, and psychological state. Moreover, some indicators have little effect on the score, such as racial factors [23]. This systematic review shows that Bufei granule causes a statistically significant improvement in the SGRQ score as compared to the placebo. These outcomes seemed promising and encouraging of the combination treatment of Bufei granule and conventional treatment to help improve lung function.

This study had some limitations. First, although the quality of the randomized controlled trials included in the meta-analysis was high, pulmonary function test indicators and the number of trials evaluated were limited. The conclusion needs further verification. At the same time, the sensitivity analysis of the SGRQ score was performed by a one-by-one elimination. This could check the stability and reliability of the results. When the study by Guo et al. [15] was excluded, the results of the meta-analysis were reversed and their conclusions would need further verification. Second, there are only few articles on Bufei granule treatment for stable COPD published in English journals. Only one article was collected in this study. Its limited evaluation outside China has affected the external effectiveness of Bufei granule treatment of stable COPD. Although this study has these limitations, it has potential significance. Bufei granule treatment can be effectively used in the intervention of stable COPD.

## 5. Conclusions

TCM Bufei granule treatment therapy may be associated with the improvement of lung function and better quality of life in COPD patients. Bufei granule could become a new treatment strategy in the intervention of stable COPD.

## Figures and Tables

**Table 1:** Description of selected studies.

Study	Sample size		Age		Interventions		Intervention time	Indicators
	Treatment	Control	Treatment	Control	Treatment	Control		
Sun et al. [18]	69	61	60.81 ± 8.18	60.51 ± 11.03	Bufei granule	Placebo	30 days	SGRQ score
Guo et al. [17]	136	135	61.89 ± 5.56	60.09 ± 5.09	Bufei granule	Placebo	12 weeks	FEV_1_; FVC; FEV_1_/predicted value; FEV_1_/FVC; SGRQ score
Yang [19]	34	34	67.7	66.5 ± 7.8	Bufei granule	Placebo	12 weeks	FEV_1_; FVC; FEV_1_/predicted value; FEV_1_/FVC; SGRQ score
Guo et al. [15]	69	61	NA	NA	Bufei granule	Placebo	12 weeks	FEV_1_; FVC; FEV_1_/predicted value; FEV_1_/FVC; SGRQ score

Abbreviations: NA, data not available; SGRQ, St George's Respiratory Questionnaire; FEV_1_, forced expiratory volume in one second; FVC, forced vital capacity.

## Supplementary Materials

Supplementary Materials Table S1: search strategy. Table S2: the PRISMA checklist. Figure S1: the funnel plot for publication bias.
