# Supplementary material for: Effect of Traditional Chinese Medicine Bufei Granule on Stable Chronic Obstructive Pulmonary Disease: A Systematic Review and Meta-Analysis Based on Existing Evidence
Source: Evid Based Complement Alternat Med. 2020 Feb 7;2020:3439457. doi: 10.1155/2020/3439457 (PMC7029284; doi:10.1155/2020/3439457)

**Table S1** Search strategy

| **Table S1**. Search strategy |
| --- |
| 1. Pulmonary Disease, Chronic Obstructive[MeSh] 2. COPD[Title/Abstract] OR Chronic Obstructive Pulmonary Disease[Title/Abstract] OR COAD[Title/Abstract] OR Chronic Obstructive Airway Disease[Title/Abstract] OR Chronic Obstructive Lung Disease[Title/Abstract] OR Airflow Obstruction, Chronic[Title/Abstract] OR Airflow Obstructions, Chronic[Title/Abstract] OR Chronic Airflow Obstructions[Title/Abstract] OR Chronic Airflow Obstruction[Title/Abstract] 3. #1OR#2 4. Bufei granule [Title/Abstract] OR Bufei keli[Title/Abstract] 5. Random* 6. #3 AND #4 AND 5# |

**Table S2** The PRISMA checklist

| **Section/topic** | **#** | **Checklist item** | **Reported on page #** |
| --- | --- | --- | --- |
| **TITLE** | | |  |
| Title | 1 | Effect of traditional Chinese medicine Bufei granule on stable chronic obstructive pulmonary disease: A systematic review and meta-analysis based on the existing evidence. | Title |
| **ABSTRACT** | | |  |
| Structured summary | 2 | Purpose: This systematic review and meta-analysis was conducted to evaluate the effect of traditional Chinese medicine (TCM)Bufei granule on stable chronic obstructive pulmonary disease(COPD).  Methods: We retrieved Pubmed, Embase, the Cochrane Central Register of Controlled Trials,Web of Science,Wanfang,CNKI and WeiPu (VIP) for studies focusing on whether the TCM Bufei granule could treat stable COPD. No blinding and language restriction was used. All trials included were analyzed according to the criteria of the Cochrane Handbook.Review Manager 5.3 software was used for data analysis.  Results: We included four studies involving 599 patients with stable COPD. Compared to placebo treatment, TCM Bufei granule treatment showed improvement in forced expiratory volume in one second (FEV1) (SMD = 0.70, from 0.50 to 0.91, I2 = 0%), forced expiratory volume(FEV) (SMD=0.43,from 0.23 to 0.62, I2 = 0%), FEV1 percentage of predicted value(FEV1(%))(SMD = 0.57, from 0.38 to 0.75, I2 = 4%), and FEV1/FVC (SMD=0.69,from 0.50 to 0.87, I2 = 0%).There was statistically significant difference in SGRQ score(SMD=-1.29,from -2.32 to-0.26, I2 = 97%) found with TCM Bufei granule compared to placebo.All studies showed no adverse events.  Conclusions: Among patients with stable COPD, TCM Bufei granule treatment therapy mightbe associated with improvement of lung function, and better quality of life. | Abstract |
| **INTRODUCTION** | | |  |
| Rationale | 3 | Chronic obstructive pulmonary disease (COPD) is a major public health problem throughout the world. In light of evidence-based medicine, short-acting bronchodilators, long-acting bronchodilators, inhaled glucocorticosteroids, and low-dose, slow-release theophylline are the established interventions for treating COPD patients. However, it is difficult to improve people’s symptoms without suffering too many side effects or adverse events. Unwanted side efects may lead some people with COPD to explore other treatment options. Therefore, designing strategies and treatments for COPD is important. Several clinical trials have shown that TCM Bufei granule might have therapeutic effect for COPD patients including improvement of quantity of life and lung function. However, the quality of these trials has not been assessed systematically. The results of these clinical trials remain controversial and inconclusive.The objective of this review was to assess positive effects of TCM Bufei granule treatment versus placebo treatment in stable COPD in adults | Introduction |
| Objectives | 4 | To assess positive effects of TCM Bufei granule treatment versus placebo treatment in stable COPD in adults. | Abstract |
| **METHODS** | | |  |
| Protocol and registration | 5 | None |  |
| Eligibility criteria | 6 | 1)randomized controlled studies on Bufei granule treat stable COPD.2) COPD patients who were diagnosed according to the diagnosis criteria in global strategy for the diagnosis, management, and prevention of chronic obstructive pulmonary disease reported by Global Initiative for Chronic Obstructive Lung Disease(GOLD); Pulmonary function test: FEV1/forced vital capacity (FVC)%<70%, 30%≤FEV1/predicted value (%)≤80%.3) the therapy of the treatment group was conventional therapy and Bufei granule.4) the therapy of the control group was conventional therapy and placebo granule.5) Outcomes of our study included George's respiratory questionnaire (SGRQ) score,FEV1,FVC,FEV1/predicted value and FEV1/ FVC. | Methods |
| Information sources | 7 | Randomized controlled studies reported through September 9 2019 were systematically searched in Pubmed, Embase, the Cochrane Central Register of Controlled Trials , Web of Science ,Wanfang, CNKI and VIP. | Methods |
| Search | 8 | Pubmed:  #1. Pulmonary Disease, Chronic Obstructive[MeSh]  #2. COPD[Title/Abstract] OR Chronic Obstructive Pulmonary Disease[Title/Abstract] OR COAD[Title/Abstract] OR Chronic Obstructive Airway Disease[Title/Abstract] OR Chronic Obstructive Lung Disease[Title/Abstract] OR Airflow Obstruction, Chronic[Title/Abstract] OR Airflow Obstructions, Chronic[Title/Abstract] OR Chronic Airflow Obstructions[Title/Abstract] OR Chronic Airflow Obstruction[Title/Abstract]  #3. #1OR#2  #4. Bufei granule [Title/Abstract] OR Bufei keli[Title/Abstract]  #5. Random*  #6. #3 AND #4 AND 5# |  |
| Study selection | 9 | Two investigators (Xinyan Wen and YiHua Fan)independently selected the studies according to the predetermined criterion using Endnote X7 software. First, duplications among the different databases were found and eliminated from the initial aggregated search results. Second, after reading the titles and abstracts, we excluded the obviously irrelevant studies. Third, we screened the full texts of the potentially relevant studies and excluded the unqualified studies. The study selection results were independently cross-checked by two investigators. Any disagreements during the study period were discussed; if the problem was not resolved, a investigator(XinYan Wen)was consulted and their decision followed. | Methods |
| Data collection process | 10 | After literature selection, two investigators (Xinyan Wen and YiHua Fan) independently extracted data from the included studies using an extraction sheet. | Methods |
| Data items | 11 | The extracted data included the name of the first author, publication year, sample size, average age of participants, time of intervention, outcome indicators, etc. We also contacted the authors via email or telephone to acquire further information when the information in the literature was incomplete. Means and standard deviations were extracted for continuous outcomes. | Methods |
| Risk of bias in individual studies | 12 | Quality of researches was assessed independently by two investigators (Xinyan Wen and YiHua Fan) using the risk of bias evaluation for randomised clinical trials in the systematic Cochrane Reviews .Evaluation items included random sequence generation, allocation concealment, blinding of participants and personnel, blinding of outcomes assessment, incomplete outcomes data, selective reporting and other biases.Third investigator(Xinju Li) resolved the discrepancies.. | Methods |
| Summary measures | 13 | The results are presented as the standardized mean difference (SMD) with the 95% confidence interval (95% CI) | Methods |
| Synthesis of results | 14 | The clinical heterogeneity of included studies was measured by the χ 2 test. If the I2 was less than 50%, we considered that the heterogeneity among studies was small, and we used the fixed effects model for data analysis. If heterogeneity was detected (I2 value ≥50%), we chose the random effects model or used only qualitative descriptions. | Methods |
| Risk of bias across studies | 15 | Egger's and Begg's test for the evaluation of potential publication bias | Methods |
| Additional analyses | 16 | The stability and reliability of the meta-analysis was evaluated by sensitivity analysis. Egger's and Begg's test for the evaluation of potential publication bias. | Methods |
| **RESULTS** |  |  |  |
| Study selection | 17 | Our search scheme yielded 154 citations, and additional 4 citations were identified through examining reference lists of relevant literatures. 15 full text were obtained after removing duplicates and reviewing abstracts, and 4 of them entered final analyses | Results |
| Study characteristics | 18 | A total of 599 patients were included in 4 studies[14, 16-18].All studies were conducted in China. The trial duration: three studies[14, 16, 18] lasted 12 weeks, and the other one studies[17] lasted 30 day | Results |
| Risk of bias within studies | 19 | The quality and the risk of bias assessment of the included studies are described in Figure 2. | Results |
| Results of individual studies | 20 | Described in Figure 3-4. | Results |
| Synthesis of results | 21 | Two studies[14, 16] reported FEV1 data. Total meta analyses showed better effect of Bufei granule than placebo (SMD = 0.70, from 0.50 to 0.91, I2 = 0%). Two studies[14, 16] used FEV as the outcome, the pooled analysis showed a statistically significant improvement in through FEV with TCM Bufei granule compared to placebo (SMD=0.43,from 0.23 to 0.62, I2 = 0%). Three studies[14, 16, 18] reported FEV1(%) data. Total meta analyses showed better effect of Bufei granule than placebo (SMD = 0.57, from 0.38 to 0.75, I2 = 4%). Three studies[14, 16, 18] used FEV1/FVC as the outcome, the pooled analysis showed a statistically significant improvement in through FEV1/FVC with TCM Bufei granule compared to placebo (SMD=0.69,from 0.50 to 0.87, I2 = 0%).Figure 3 shows the efficacy of Bufei granule for stable COPD. Four studies[14, 16-18] used the SGRQ, a decrease in SGRQ score denotes an improvement in quality of life. TCM Bufei granule led to a statistically significant improvement in health-related quality of life compared to placebo (SMD=-1.29,from -2.32 to-0.26, I2 = 97%) | Results |
| Risk of bias across studies | 22 | Described in Figure 2. | Results |
| Additional analysis | 23 | The stability and reliability of the meta-analysis was evaluated by sensitivity analysis. For SGRQ score, when the study (Guo et al)[14] was excluded, the result was reversed, indicating that the study result was not stable. Other outcome measures were stability and reliability.The results showed no statistically significant difference, Begg's Test(P= 0.497),Egger's test(P= 0.338). | Results |
| **DISCUSSION** |  |  |  |
| Summary of evidence | 24 | This systematic review shows the current evidence in Bufei granule for COPD. Bufei granule plus conventional medicine treatment was associated with improvement of lung function, and better quality of life in COPD patients with the treatment of placebo plus conventional medicine | Discussion |
| Limitations | 25 | Firstly, although the quality of CT included in Meta analysis is very high, pulmonary function testing indicators and the number of RCT evaluated are limited. Its conclusion needs further verification. At the same time, the sensitivity analysis of SGRQ score is carried out by eliminating one by one. This can check the stability and reliability of the results. When Guo et al[14]. are excluded from the study, the results of meta-analysis are reversed, and their conclusions need to be further verified. Secondly, there are fewer articles on Bufei Granule treatment of stable COPD published in English journals. Only one article is collected in this study. Its limited evaluation outside China has affected the external effectiveness of Bufei Granule in the treatment of stable COPD. Although this study has some limitations, it has potential significance. Bufei Granule has an advantage in the treatment of stable COPD. | Discussion |
| Conclusions | 26 | Among patients with stable COPD, TCM Bufei granule treatment therapy mightbe associated with improvement of lung function, and better quality of life.Bufei granule could be a new option in the treatment of stable COPD. | Conclusions |
| **FUNDING** |  |  |  |
| Funding | 27 | This research was supported by National Basic Research Program of China under Grant No. 2014CB543201. The funding source had no role in the design of this study and will not have any role during its execution, analyses, interpretation of the data, or decision to submit results. |  |

**Figue S1** The funnel plot for publication bias


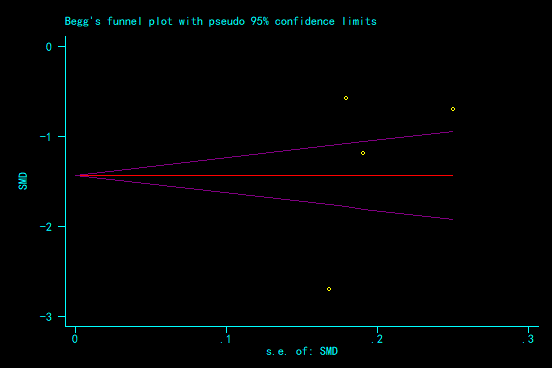

Supplement: Supplementary Materials — Table S1: search strategy. Table S2: the PRISMA checklist. Figure S1: the funnel plot for publication bias. [file 3439457.f1.docx]
